# Short Term Recovery of Function following Total Knee Arthroplasty: A Randomised Study of the Medial Parapatellar and Midvastus Approaches

**DOI:** 10.1155/2014/173857

**Published:** 2014-10-01

**Authors:** Richard W. Nutton, Frazer A. Wade, Fiona J. Coutts, Marietta L. van der Linden

**Affiliations:** ^1^Royal Infirmary, 51 Little France Crescent, Edinburgh EH16 4SA, UK; ^2^Rehabilitation Sciences, Queen Margaret University, Queen Margaret University Drive, Musselburgh EH21 6UU, UK

## Abstract

This pilot double blind randomised controlled study aimed to investigate whether the midvastus (MV) approach without patellar eversion in total knee arthroplasty (TKA) resulted in improved recovery of function compared to the medial parapatellar (MP) approach. Patients were randomly allocated to either the MV approach or the MP approach. Achievements of inpatient mobility milestones were recorded. Knee kinematics, muscle strength, Timed Up and Go, WOMAC, and daily step count were assessed before and up to six months after surgery. Cohen's effect size *d* was calculated to inform the sample size in future trials. Twenty-eight participants (16 males, 12 females) participated. Patient mobility milestones such as straight leg raise were achieved on average 1.3 days (95% CI −3.4 to 0.7, d = 0.63) earlier in the MV group. Knee extensor strength at 6 weeks after surgery was higher (95% CI −0.38 to 0.61, d = 0.73) in the MV group. No trends for differences between the groups were observed in knee kinematics, TUG, WOMAC, or step count. Our results suggest a short term advantage in the first 6 weeks after surgery of the MV approach over the MP approach, but a larger study is required to confirm these findings. This trial is registered with NCT056445.

## 1. Introduction

Total knee replacement surgery for osteoarthritis has been shown to be successful in relieving pain and improve function and quality of life for the majority of patients. However, continued efforts are directed to further optimise the functional outcome of total knee surgery by attempting to improve implant design [1, 2] and more recently the type of surgical approach used. The term “minimally invasive surgery” covers a variety of approaches including midvastus and mini-parapatellar approaches with or without computer navigation. Several authors have compared the effects on outcome of the midvastus (MV) approach compared to the more traditional medial parapatellar (MP) approach and their results have been summarised by reviews [3–5] concluding some early benefits on mobility milestone and muscle strength. However, only few studies [6–9] included in the above reviews and more recent studies [10, 11] were prospective randomised controlled trials and only few reported a double blind design. A recent study comparing the midvastus approach with the medial parapatellar approach in bilateral TKA, by Nestor et al. [10], was the only one to standardise incision length allowing both the patient and the assessor to be blind to group allocation.

Jarvis et al. [12] compared the knee kinematics and kinetics during sit-to-stand transitions in patients who had received either the standard parapatellar surgical approach (SP) or mini-parapatellar (MP) surgical approach performed using computer navigation. However, to the authors' knowledge no double blind randomised controlled studies have investigated whether the recovery of gait kinematics differs between patients undergoing surgery using either the MV approach or the MP approach.

Therefore, the present pilot study was designed as a double blind randomised controlled trial to assess prospectively functional outcomes in the first six months following unilateral total knee arthroplasty using either a medial parapatellar approach or a midvastus approach to the knee. We hypothesised that the midvastus (MV) approach without patellar eversion would reduce damage to the quadriceps compared to the medial parapatellar (MP) approach and this would result in improved recovery of function such as gait kinematics. In addition to knee kinematics during walking, we evaluated the outcomes in all three domains of the International Classification of Health (ICF) [13], “Body Structures and Function” (e.g., range of motion and muscle strength), “Activity” (e.g., gait kinematics and Timed Up and Go test), and Participation' (daily step count).

## 2. Patients and Materials

Patients were recruited to the study from the waiting list of two consultants with a special interest in knee arthroplasty. Patients included were those with knee osteoarthritis and on the waiting list for primary unilateral knee arthroplasty and those who are fit enough to take part in the gait analysis assessments. Exclusion criteria were a body mass index of more than 40; a fixed valgus deformity of more than 15 degrees; inflammatory polyarthritis; disorders of the feet, ankles, hips, or spine causing abnormal gait or significant pain; dementia; severe visual impairment; neurological conditions affecting movement; and the inability to give informed consent. Patients who had previously had hip replacements or contralateral knee replacements were not excluded, unless they had on-going pain or restricted function.

Ethical approval was granted from the local hospital trust and the University Research Ethics Committees. All participants gave informed consent, signing a consent form before participating in the study.

The study has been registered with ClinicalTrials.gov (NCT056445).

### 2.1. Surgical Protocols

Knee arthroplasties were performed using either the medial parapatellar approach or midvastus approach. A standard 15 cm skin incision was used in all cases so that it was not possible for the patient or the research assessor to detect which approach had been used. PFC Sigma cruciate retaining arthroplasty (DePuy International Ltd, Leeds, UK) implants were used with cement and the patella was not resurfaced. All patients had surgery under spinal anaesthesia supplemented by a femoral nerve block. All femoral nerve blocks were undertaken with a nerve stimulator and 20 mL of 0.25% marcaine infiltrated. They were all effective and resulted in less than 24 hours of motor blockade. In the postoperative period, patient controlled analgesia was used for 24 hours. The postoperative care for both of the patient groups was identical, using a standard care plan which emphasizes early weight bearing and knee flexion exercises. Discharge from hospital was dictated by individual patient's ability to ascend and descent a flight of stairs and home circumstances.

Participants were randomly allocated to either the “medial parapatellar” (MP) group or the “midvastus” (MV) group using computer generated randomization. Cards instructing “medial parapatellar” group or “midvastus” group were placed in opaque sealed envelopes which were opened when the patient was prepared and draped for surgery, immediately before proceeding with the operation.

### 2.2. Outcome Measures

Patients in the study were assessed on average 26 days before being admitted for a knee replacement surgery and at six weeks, three months, and six months after surgery. Functional assessments were performed by a researcher who had no involvement with the patient's care and thus was blinded to group allocation. Tourniquet time was recorded. The first postoperative day the patients achieved their mobility milestones (straight leg raise, progression from walking with a Zimmer frame to walking with two sticks and independent stair climbing) was also recorded.

### 2.3. Laboratory Outcome Measures

The knee extensor force was measured before surgery and at six-week, three-month, and six-month postoperative assessments, using MIE myometer (MIE, Leeds, UK) with the participants sitting on a chair with their knee in 90 degree flexion. The peak moment calculated by multiplying the force value by the moment arm was normalized by dividing by body weight. The maximum value over three trials was used for analysis.

Active peak flexion and extension of the affected knee were measured with participants being seated on a plinth. A manual goniometer was used to measure the angle to the nearest degree.

The Timed Up and Go test (TUG) records the time taken to rise from a standard arm chair, walk to a line three metres from the chair, turn around, walk back, and sit down on the chair again [14].

Three-dimensional motion analysis using eight camera Vicon Nexus systems was used to record the lower limb kinematics before surgery and at the three-month and six-month postoperative assessments. Participants had 14 mm diameter passive reflective sphere markers placed on their lower limbs and pelvis according to the Vicon Plug-In-Gait manual which is based on the Helen Hays marker system [15]. Participants were asked to walk a distance of six metres across the laboratory while the Vicon motion analysis system was recording; this constituted one trial. A minimum of five trials were recorded for each assessment.

The following parameters were retrieved for analysis: walking speed, peak knee flexion in swing, peak knee extension in stance, knee range of motion during the stance phase (load acceptance), and knee and hip range of motion over the entire gait cycle.

### 2.4. Patient Reported Outcomes and Activity Monitoring

Patients were asked to complete the Western Ontario and McMaster Universities Osteoarthritis Index (WOMAC). Daily physical activity was estimated using an activPAL activity monitor [16]. Participants were given the activity monitor at the presurgery and the six-month assessments and were asked to wear the monitor for a minimum of five days, including at least one day over the weekend.

### 2.5. Statistical Analysis

An independent *t*-test or the Mann-Whitney *U* test for nonparametric data was used to compare differences between the two groups before surgery. Differences between the two groups were analysed using repeated measures two-way analysis of variance (ANOVA). To account for possible differences between the groups at baseline preoperative measures were used as covariant. Level of statistically significant difference was set at *P* < 0.05.

Cohen's effect size *d* was calculated to inform sample size for future, appropriately powered, larger scale trials. Effect sizes were defined to be medium for values for Cohen's *d* of more than 0.3 but less than 0.5, good for values of 0.5 and greater but less than 0.8, and large for values of 0.8 and greater [17]. Statistical calculations were performed using Statistical Package for Social Sciences (SPSS), version 19.

## 3. Results

**Figure 1:**
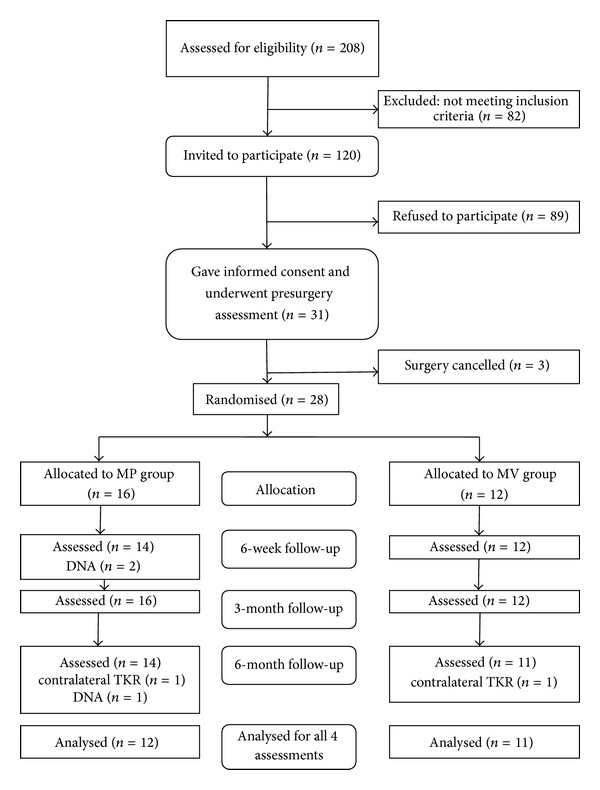
Consort flow diagram of the study.

Two hundred and eight patients with osteoarthritis were identified and after application of the exclusion criteria 120 were invited to take part in the study, 31 of whom attended for the preoperative assessment. Of those 31 patients, the surgery of three patients was cancelled, resulting in a total of 28 patients randomized to either approach. Sixteen had a medial parapatellar approach and 12 had a midvastus approach. The CONSORT flow diagram is included in Figure 1.

### 3.1. Effects of Surgical Approach

A comparison of the two groups for participants' demographic and baseline characteristics is shown in Table 1. There were no statistically significant differences between the MP and MV groups with regard to age, gender, and preoperative range of motion.

#### 3.1.1. Hospital Outcomes Recorded within a Week from Surgery

Outcomes recorded during hospital stay are shown in Table 2. Tourniquet time was significantly shorter in the MV group (49 versus 61 minutes, *P* = 0.029), but when surgeon was used as covariate, this difference was not statistically significant (*P* = 0.1).

The MV group showed up to 5 degrees more ROM in the first, second, and third days after surgery and achieved their mobility milestones earlier. Straight leg raise was achieved 1.3 days earlier (95% Confidence Interval: −3.4 to 0.7, Cohen's *d* = 0.63), progression from a walker to two walking sticks was achieved 0.8 days earlier (CI: −2.3 to 0.7, Cohen's *d* = 0.55), and stair negotiation was achieved 0.9 days earlier (CI −2.8 to 1.0, Cohen's *d* = 0.50) in the MV group compared to the MP group. However, although possibly clinically relevant with good effect sizes (Cohen's *d* ≥ 0.5), none of these advantages of the MV approach over the MP approach were statistically significant in this pilot study. Interestingly, although the clinical pathway states that patients are ready to be discharged once they can safely negotiate a flight of stairs, unlike days to stair negotiation, days to discharge did not show a trend towards an advantage of the MV approach (5.3 days versus 5.2 days; Cohen's *d* = 0.04).

#### 3.1.2. Laboratory and Self-Reported Outcomes

**Figure 2:**
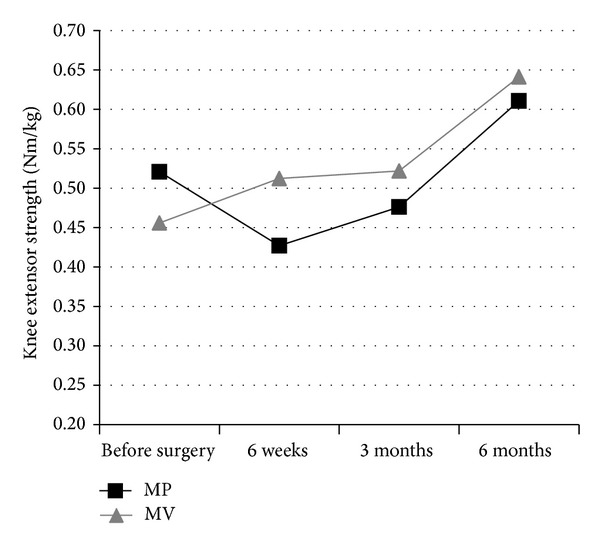
Graphical representation of the recovery of the knee extensor strength in the midvastus (MV) group and the medial parapatellar (MP) group.

Table 3 shows the results of the measurements of knee extensor strength, active knee flexion, TUG, and the WOMAC. Knee extensor strength was lower relative at the six week post surgery assessment, knee extensor strength had decreased compared to before surgery in the MP group, while in the MV group knee extensor strength was slightly improved (changes: MP −0.09 Nm/kg versus MV 0.06 Nm/kg, Cohen's *d* = 0.73, *P* = 0.269).  Figure 2 shows the recovery profiles of extensor strength. No differences between the groups in the rate of improvement of active knee flexion or TUG were observed.

Knee and hip kinematics and daily step count are shown in Table 4. None of these outcomes showed statistically or clinically significant differences between two groups.

## 4. Discussion

The purpose of this study was to detect differences in the recovery of knee function following knee replacement surgery using two different approaches, the medial parapatellar and the midvastus without patellar eversion. The assessments focused on functional outcome measures during the recovery from knee replacement in the first six months. We did not attempt to compare pain medication levels in the two groups which has been the subject of previous studies [18, 19] investigating the effects of surgical approach.

The results of this study suggest that the MV group showed earlier achievement of mobility milestones such as straight leg raise, progression from a walker to two walking sticks, and independent stair negotiation. The difference between surgical approaches with regard to “days to straight leg raise” found in our study (1.3 days) is similar to previous studies reporting statistically significant differences of 1.1 days [20] and 1.5 days [21] in favour of the MV approach. White et al [22] also reported that patients who underwent a MV approach were more likely to be able to achieve a straight leg raise at 8 days post-surgery compared to MP approach. In addition, the current study showed a trend towards quicker recovery of the muscle function in the MV group. On average, participants in the MV group had increased their knee extensor strength at six weeks and three months relative to before surgery, while those in the MP group showed a decrease in strength at those time points. Nestor et al [10] reported improved reported strength three weeks after surgery in participants who underwent the MV approach compared to the MP approach, but this benefit did not last at six and twelve weeks after surgery.

Early knee flexion showed small advantages in the MV group participants over the MP group participants. Mean peak flexion over the first three days was 5 degrees higher in the MV group. In comparison, the meta-analysis by Alcelik et al. [4] showed significantly higher differences in flexion at one week. (8.88 degrees, 95% confidence interval (CI) 4.50 to 13.25, *P* < 0.01) in favour of the MV approach, while Karachalios et al. [9] reported differences in favour of the midvastus approach ranging from 51 days at day 1 to 32 degrees at day 21. The meta-analysis by Liu and Yang [3] also showed higher knee ROM at day 1 and at three months after surgery.

Functional outcomes measured by the WOMAC score, knee ROM assessed by gait analysis and daily physical activity at three and six months measured by activPAL, did not show any benefits of the MV approach. The lack of an effect of comparing the MV and MP approaches on functional outcomes such as the KSS was also reported in a meta-analysis by Alcelik et al. [4] who did however conclude that pain in the first week after surgery was reduced in the MV group relative to the MP group. Pain scores in the first week, however, are difficult to compare amongst different studies as this highly depends on the pain medication protocols in the first days after surgery.

This study has several limitations. Firstly, the current study was a pilot study and the limited number of participants negatively impacted the power to detect statistically significant differences between the groups. Secondly, the participants in the trial were only a small proportion of those undergoing surgery at our hospital during the study period (31/208, 14.9%), which limits the generalizability of the results of the study. Also, two surgeons performed the surgery in this trial which may introduce heterogeneity of the data. Finally, knee kinematics during walking were not recorded at 6 weeks after surgery as it was thought that this may be too demanding for participants. However, this may have provided more insights into objective function in the early stages of the recovery.

To the authors' knowledge, this current randomized controlled trial is the first study investigating the difference in knee kinematics of unilateral TK between the MV approach and MP approach which allowed double blinding because of similar incision length in both approaches. In addition, the effect of surgical approach in this pilot study was assessed on a whole range of outcome measures in all three domains of the ICF. Objective outcomes of function such gait analysis were assessed in addition to outcomes such as achievement of mobility milestones and days until discharge which are important from the health economics point of view.

Although surgical approach did not appear to affect function past 6 weeks post surgery, good effect sizes found for outcomes in the first 6 weeks indicated a benefit of the MV approach regarding the achievement of early mobility milestones and in the early recovery of muscle strength without compromising on surgical duration. As these trends confirmed by results from previous studies are of interest for both patients' benefit and the health economic point of view, a future appropriately powered trial with a similar design but including a health economic analysis is warranted. Based on the effect sizes in this pilot study, a power calculation for such a future trial suggest that 85 participants in each group would be required to detect an effect size of 0.5 (days until patient meet physiotherapy discharge criteria) or 41 in each group to detect an effect size of 0.73 (knee extensor strength at 6 weeks). In addition, further investigations should be carried out into how the apparently early advantage of the MV approach can be maintained for a longer term.

## Figures and Tables

**Table 1:** Mean (std) values of the preoperative measures for the medial parapatellar (MP) and midvastus (MV) groups.

	Intention to treat			Per protocol		
	MP (*n* = 16)	MV (*n* = 12)	*P* value	MP (*n* = 12)	MV (*n* = 11)	*P* value
Age (yrs)	70 (8)	73 (8)	0.227	71 (7)	74 (8)	0.371
Gender (male : female)	8/8	8/4	0.209^*¥*^	7/5	7/4	0.681^*¥*^
BMI (kg/m^2^)	29.3 (3.4)	31.1 (4.4)	0.240	28.9 (2.6)	31.2 (4.3)	0.056
TUG (s)	11.5 (4.1)	11.3 (2.9)	0.874	10.5 (2.3)	11.5 (2.9)	0.406
Active flexion (°)	111 (13)	106 (19)	0.434	109 (13)	105 (18)	0.519
Extensor strength (Nm/kg)	0.50 (0.21)	0.48 (0.18)	0.808	0.52 (0.22)	0.45 (0.18)	0.533
WOMAC pain (max 20)∗	9.4 (3.3)	8.7 (3.1)	0.537	9.4 (3.2)	8.6 (3.1)	0.427
WOMAC stiffness (max 8)∗	4.8 (1.2)	3.8 (1.5)	0.048	4.8 (1.8)	3.9 (1.5)	0.054
WOMAC function (max 96)∗	31.2 (9.4)	28.0 (10.3)	0.402	30.3 (10.1)	27.1 (11.0)	0.495

*A higher value means more pain, more stiffness, and a decreased function.

^*¥*^Fisher's exact test.

**Table 2:** Mean (std) values of the in-patient postoperative measures for the medial parapatellar (MP) and midvastus (MV) groups. *P* value of independent *t*-tests unless stated otherwise.

	MP	MV	*P* value	Difference and 95% CI
Tourniquet time (min)	61 (15)	49 (8)	**0.029**	**11 ** **(1 : 22)**
Active flexion day 1 (°)	39 (14)	43 (17)	0.582	4 (−9 : 16)
Active flexion day 2 (°)	56 (17)	61 (18)	0.470	−5 (−9 : 18)
Active flexion day 3 (°)	70 (19)	75 (16)	0.491	−5 (−10 : 20)
Days until straight leg raise	3.9 (2.7)	2.6 (1.4)	0.338^¥^	−1.3 (−3.4 : 0.9)
Days until two walking sticks	4.0 (2.3)	3.2 (0.6)	0.605^¥^	−0.8 (−2.3 : 0.7)
Days until stair negotiation	5.0 (2.7)	4.1 (1.3)	0.461^¥^	−0.9 (−2.8 : 1.0)
Length of hospital stay	5.3 (2.8)	5.2 (2.9)	0.978^¥^	−0.1 (−2.5 : 2.2)

^*¥*^Nonparametric Mann-Whitney *U* test.

**Table 3:** Mean (std) values of the pre- and postoperative function measures for the MP and MV groups. *P* value for the effect of the type of surgery.

		Before surgery	6 weeks	3 months	6 months	*P* value
Knee extensor strength (Nm/kg)	MP	0.52 (0.22)	0.43 (0.13)	0.48 (0.09)	0.61 (0.25)	0.269
	MV	0.45 (0.18)	0.51 (0.25)	0.52 (0.23)	0.64 (0.25)	

Active flexion (°)	MP	109 (13)	95 (15)	103 (6)	104 (7)	0.283
	MV	105 (18)	90 (13)	98 (12)	100 (14)	

TUG (s)	MP	10.5 (2.3)	11.0 (2.7)	9.5 (2.1)	9.5 (2.8)	0.387
	MV	11.5 (2.9)	11.5 (2.5)	11.4 (3.0)	10.5 (2.1)	

WOMAC pain (max 20)	MP	9.4 (3.2)	5.5 (2.5)	4.3 (2.8)	3.8 (3.1)∗	0.907
	MV	8.6 (3.1)	5.1 (3.6)	4.7 (4.3)	3.2 (3.6)	

WOMAC stiffness (max 8)	MP	4.8 (1.8)	2.8 (1.8)	2.7 (1.7)	2.5 (1.4)	0.799
	MV	3.9 (1.5)	2.7 (1.5)	1.9 (1.2)	2.6 (1.3)	

WOMAC function (max 96)	MP	30.3 (10.1)	16.7 (10.8)	14.4 (9.8)	14.6 (11.1)	0.964
	MV	27.1 (11.0)	14.8 (10.5)	13.9 (13.0)	13.8 (10.6)	

*Nonparametric Mann-Whitney *U* test.

**Table 4:** Mean (std) values of the pre- and postoperative gait characteristics of the medial parapatellar (MP) and midvastus (MV) groups. *P* value for the effect of surgical approach.

		Before surgery	3 months	6 months	*P* value
Walking speed (m/s)	MP	0.83 (0.18)	0.90 (0.22)	0.96 (0.15)	0.666
	MV	0.80 (0.15)	0.84 (0.21)	0.88 (0.18)	

Knee ROM Gait Cycle (°)	MP	42.8 (10.3)	41.9 (11.4)	45.8 (9.7)	0.811
	MV	43.7 (7.9)	40.4 (10.5)	43.4 (9.6)	

Knee ROM in stance (°)	MP	5.1 (3.0)	5.4 (2.9)	5.8 (2.8)	0.983
	MV	6.3 (4.0)	6.5 (3.3)	7.1 (3.2)	

Peak extension stance (°)	MP	8.8 (9.2)	8.4 (5.8)	6.3 (7.6)	0.599
	MV	7.0 (6.4)	8.6 (5.2)	3.7 (6.5)	

Hip ROM gait cycle	MP	36.9 (8.4)	39.1 (8.1)	40.0 (7.1)	0.670
	MV	35.7 (4.3)	36.3 (8.2)	38.3 (6.5)	

Steps per day	MP	5613 (2505)	NM	5786 (1916)	0.126
	MV	6342 (2695)	NM	6822 (2363)	
